# Artificial Intelligence versus Statistical Modeling and Optimization of Cholesterol Oxidase Production by using *Streptomyces* Sp.

**DOI:** 10.1371/journal.pone.0137268

**Published:** 2015-09-14

**Authors:** Lakshmi Pathak, Vineeta Singh, Ram Niwas, Khwaja Osama, Saif Khan, Shafiul Haque, C. K. M. Tripathi, B. N. Mishra

**Affiliations:** 1 Department of Biotechnology, Institute of Engineering and Technology (Uttar Pradesh Technical University), Lucknow, 226021, India; 2 Microbiology Division, CSIR-Central Drug Research Institute, Sitapur Road, Lucknow, 226031, Uttar Pradesh, India; 3 Deratment of Clinical Nutrition, College of Applied Medical Sciences, Ha’il University, Ha’il, Saudi Arabia; 4 Centre for Drug Research, Faculty of Pharmacy, Viikki Biocenter-2, University of Helsinki, Helsinki, FIN-00014, Finland; 5 Research and Scientific Studies Unit, College of Nursing & Applied Health Sciences, Jazan University, Jazan, 45142, Saudi Arabia; 6 Fermentation Technology Division, CSIR-Central Drug Research Institute, Sitapur Road, Lucknow-226031, Uttar Pradesh, India; Laurentian University, CANADA

## Abstract

Cholesterol oxidase (COD) is a bi-functional FAD-containing oxidoreductase which catalyzes the oxidation of cholesterol into 4-cholesten-3-one. The wider biological functions and clinical applications of COD have urged the screening, isolation and characterization of newer microbes from diverse habitats as a source of COD and optimization and over-production of COD for various uses. The practicability of statistical/ artificial intelligence techniques, such as response surface methodology (RSM), artificial neural network (ANN) and genetic algorithm (GA) have been tested to optimize the medium composition for the production of COD from novel strain *Streptomyces* sp. NCIM 5500. All experiments were performed according to the five factor central composite design (CCD) and the generated data was analysed using RSM and ANN. GA was employed to optimize the models generated by RSM and ANN. Based upon the predicted COD concentration, the model developed with ANN was found to be superior to the model developed with RSM. The RSM-GA approach predicted maximum of 6.283 U/mL COD production, whereas the ANN-GA approach predicted a maximum of 9.93 U/mL COD concentration. The optimum concentrations of the medium variables predicted through ANN-GA approach were: 1.431 g/50 mL soybean, 1.389 g/50 mL maltose, 0.029 g/50 mL MgSO_4_, 0.45 g/50 mL NaCl and 2.235 ml/50 mL glycerol. The experimental COD concentration was concurrent with the GA predicted yield and led to 9.75 U/mL COD production, which was nearly two times higher than the yield (4.2 U/mL) obtained with the un-optimized medium. This is the very first time we are reporting the statistical versus artificial intelligence based modeling and optimization of COD production by *Streptomyces* sp. NCIM 5500.

## Introduction

The production of metabolites produce through microbial strains is mostly affected by the process parameters and medium components. Generally, the fermentation processes are multi-variable and optimization of medium components is a cumbersome task. The conventional one factor at a time (OFAT) approach is time-consuming and often incapable of reaching the true optimum due to complex interactions among the factors/ variables [1]. Generally, statistical or mathematical designs are used to reduce the number of experiments and to increase the precision of the results. Response surface methodology (RSM) is a combination of mathematical and statistical techniques and generally used for modeling and analysis of problems associated with multivariable systems. It is based on design of experiments (DOE) for the development of models, estimation of the model coefficients and prediction of the response for optimum conditions [2, 3]. RSM estimates the relationship between the responses (i.e., product yield) and the experimental parameters (i.e., concentration of the medium components). It adjusts the concentration of the medium components to shift the product yield (response) in a certain direction to achieve the required optima. The RSM has been successfully applied for optimization of medium components for metabolite production [1, 4], culture parameters in bioprocess engineering [5–7], etc. Despite its successful use in various processes, RSM has some limitations like, in case of more than six or seven variables, the number of variables interaction terms will increase and resulted in complexity of the study and the practical feasibility of the method will challenged [8]. In addition, the RSM fails to precisely describe an object function [9].

Artificial Neural Networks (ANNs) are complex mathematical models that successfully mimic biological neural networks. ANNs have been used for optimization and prediction purposes and are often preferred over regression models for the noisy data. ANNs have been used to optimize and model highly nonlinear and complex biological processes [10–18] etc. Mathematical model generated by RSM or ANNs can be optimized more precisely by using mathematical tools, like Nelder-Mead simplex, genetic algorithm (GA) etc. GA is an optimization tool which can be used even under conditions of unavailability of complete model of the process. GA is based on Darwin’s principle of genetic evolution and uses genetic operators, like selection, mutation and crossover to find the optimum solution of the problems. In terms of microbiological metabolite production process, the media components are represented as genomes or chromosomes and the factors to be optimized i.e., level of medium constituents are represented as genes [19]. The chromosomes with high productivity are selected and replicated proportionally to the productivity. GA randomly selects the individuals, from the current population and uses them to produce the next generation. Over successive generations, the population “evolves” toward an optimal solution.

Cholesterol oxidase (COD; cholesterol: oxygen oxidoreductase, EC 1.1.3.6), a bi-functional FAD-containing enzyme belongs to the family of oxidoreductases and catalyzes the oxidation of cholesterol into 4-cholesten-3-one in the presence of O_2_ and isomerization of 4-cholesten-3-one into Δ^4^-3-ketosteroid [20]. COD has received great importance due to its broad application in clinical laboratories for the determination of serum cholesterol, used as a biocatalyst for the production of various steroids, and implicated in the manifestation of some bacterial and viral diseases. These biotechnological applications COD have warranted for screening, isolation and characterization of newer microbes from diverse habitats as a source of COD and optimization and microbial COD production at commercial scale [20, 21]. This study attempts to determine the quantitative effects of five medium components (soybean meal, glycerol, maltose, sodium chloride and magnesium sulphate) on COD production by *Streptomyces* sp. NCIM 5500 using statistical Response Surface Methodology and artificial intelligence technique followed by optimization using Genetic Algorithm

COD production by *Streptomyces* sp. NCIM 5500 was studied under different production media *viz*. Cholesterol enrichment medium, MGYP medium, X-medium and YMG medium [22]. Cholesterol enrichment medium and X- medium were found to be the best producers of COD [22]. In order to keep the production cost effective and economical, soybean meal based X-medium was selected for the production and optimization of COD in the present study.

## Materials and Methods

### Microbial strain and fermentation conditions

The COD producing microbial strain was isolated from pre-treated soil sample collected from the agricultural fields of Northern India as reported earlier [22]. The strain was characterized on the basis of 16S rRNA homology (Gene Ombio Technologies, Pune, India [22]. Seed flask was prepared by inoculating (with a loop full slant culture) the medium having composition of 0.5 g/L MgSO_4_.7H_2_O, 0.5 g/L (NH_4_)_2_HPO_4_, 3 g/L NaCl, 1 g/L K_2_HPO_4_, 10 g/L soybean meal, 3 g/L CaCO_3_ and 15 ml glycerol. The culture was incubated at 28°C for 48 h at 180 rpm. Two percent (v/v) inoculum was used to inoculate the production medium with the similar composition as mentioned above for the seed medium. For the production of enzyme, the flasks were incubated at 28°C for 96 h at 180 rpm.

### Enzyme assay and protein estimation

The culture broth was centrifuged at 10,000 rpm for 15 min at 4°C and the supernatant was used as a source of COD. The enzymatic activity of COD was assayed by Allain’s method of cholesterol conversion into 4-cholesten-3-one [23]. For the assay, 3.03 mL reaction mixture was prepared comprising of 94 mM potassium phosphate, 0.35% Triton X-100, 3.4 mM taurocholic acid, 0.9 mM cholesterol, 19.8 mM phenol, 1.5mM 4-aminoantipyrine and 19 units of horse radish peroxidase (HRP) enzyme isolated from horseradish root (*Amoracia rusticana)*. The reaction mixture was incubated at 37°C for 5 min afterwards it was boiled for 5 min in a water-bath to stop the reaction. The reaction mixture was cooled at room temperature and the absorbance was measured at 500 nm. One unit of COD is defined as the amount of enzyme required to produce 1 μmol of 4-cholesten-3-one per min under the test condition. Total protein concentration in the broth was determined by Lowry’s method using bovine serum albumin (BSA) as a standard [24].

### Selection of effective medium components

The most suitable production medium with highest productivity was selected by observing the production of COD under different media [22]. At the end, soybean meal based X-medium was selected for further experiments related to the enhancement of COD concentration [22]. Classical approaches, like removal, supplementation and replacement experiments were performed using OFAT methodology for the selection of effective medium components for COD production [1]. All experiments were performed in triplicate and the average values were used for the calculations.

### Modeling and optimization of medium for COD production

Response surface models are multivariable polynomial models, mostly used to determine a set of variables that optimize a response (i.e., COD concentration in this study). Five medium components *viz*. soybean, glycerol, maltose, MgSO_4_ and NaCl were selected to generate the model for response optimization. The circumscribed central composite design (CCD) was used to study the interaction effect between the above mentioned variables/ factors. The uncoded and coded values of the variables at five levels of CCD have been summarized in Table 1. For five variables, thirty six run CCD design containing ten star points, ten centre points and sixteen axial points were generated by using *ccdesign* function of the statistical tool box of MATLAB 7.10.0 (R2010a) (Math Works Inc., USA). The activity of COD was estimated for each experimental run. A quadratic response surface model was generated and its polynomial coefficients were calculated using statistical tool box of MATLAB. The experimental results were fitted to the quadratic equation (Eq 1) given by *regstat* function of the statistical toolbox of MATLAB to determine the coefficients of the equation and to obtain an optimum response surface model.

**Table 1:** Independent variables and their coded and un-coded values.

Symbol	Variables	Coded value				
		-2	-1	0	+1	+2
X1	Soybean (g/50 mL)	0.0005	0.375	0.75	1.125	1.5
X2	Glycerol (mL/50 mL)	0.0005	0.375	0.75	1.125	1.5
X3	Maltose (g/50 mL)	0.0005	0.375	0.75	1.125	1.5
X4	MgSO_4_(g/50 mL)	0.0005	0,0125	0.025	0.0375	0.5
X5	NaCl (g/50 mL)	0.0005	0.075	0.15	0.225	0.3

$$Y\left(X\right)=a_{0}+\sum_{i=0}^{N}a_{i}X_{i}+\sum_{i<j}^{N}a_{ij}X_{i}X_{j}+\sum_{i=0}^{N}a_{ii}X_{i}^{2}$$ (1)

Where, *Y* is the predicted response, *a*_0_ is the intercept coefficient, *a*_*i*_ *X*_*i*_ are the linear terms, *a*_*ij*_ *X*_*i*_ *X*_*j*_ are the interaction terms and *a*_*ii*_ *X* ^*2*^ are the square terms.

Additionally, ANN was used to model the effect of the five media components on enzyme activity. Different architectures of feed forward neural network were designed and trained using neural network tool box of MATLAB. Different combinations of transfer functions were used as input and hidden layers while neurons as output layers containing ‘purelin’ transfer function. The networks were trained with a training data-set comprising 30 experimental runs (24 training runs and 6 test runs). The training of the networks was done by using three functions *viz*. gradient descent, gradient descent with adaptive learning and Levenberg-Marquardt training algorithm using MATLAB *traingd*, *traingda* and *trainlm* functions, respectively. The trained network models were simulated and validated using validation data set (experimental data which was not used for training) for precision.

The models generated through RSM and ANN were further optimized by employing genetic algorithm *ga* function of MATLAB. The input parameters of ‘*ga*’ function were as follows: Population Type: 'double Vector'; Pop Init Range: [2x1 double]; population Size: 200; elite count: 2; crossover fraction: 1; migration direction: 'forward'; migration interval: 20; migration fraction: 0.2000; generations: 100; time limit: Inf; fitness limit:-Inf; stall gen limit: 50; stall time limit: 20; initial population: []; initial scores: []; plot interval: 1; creation fcn: @gacreationuniform; fitness scaling fcn: @fitscalingrank; selection fcn: @selectionstochunif; Crossover Fcn: @crossoverscattered; mutation fcn: {[1x1 function_handle] [1] [1]}; hybridfcn: []; display: 'off'; plotfcns: {[1x1 function_handle] [1x1 function_handle]};outputfcns: []; vectorized: 'off'.

## Results

### Selection of effective medium components

**Fig 1:**
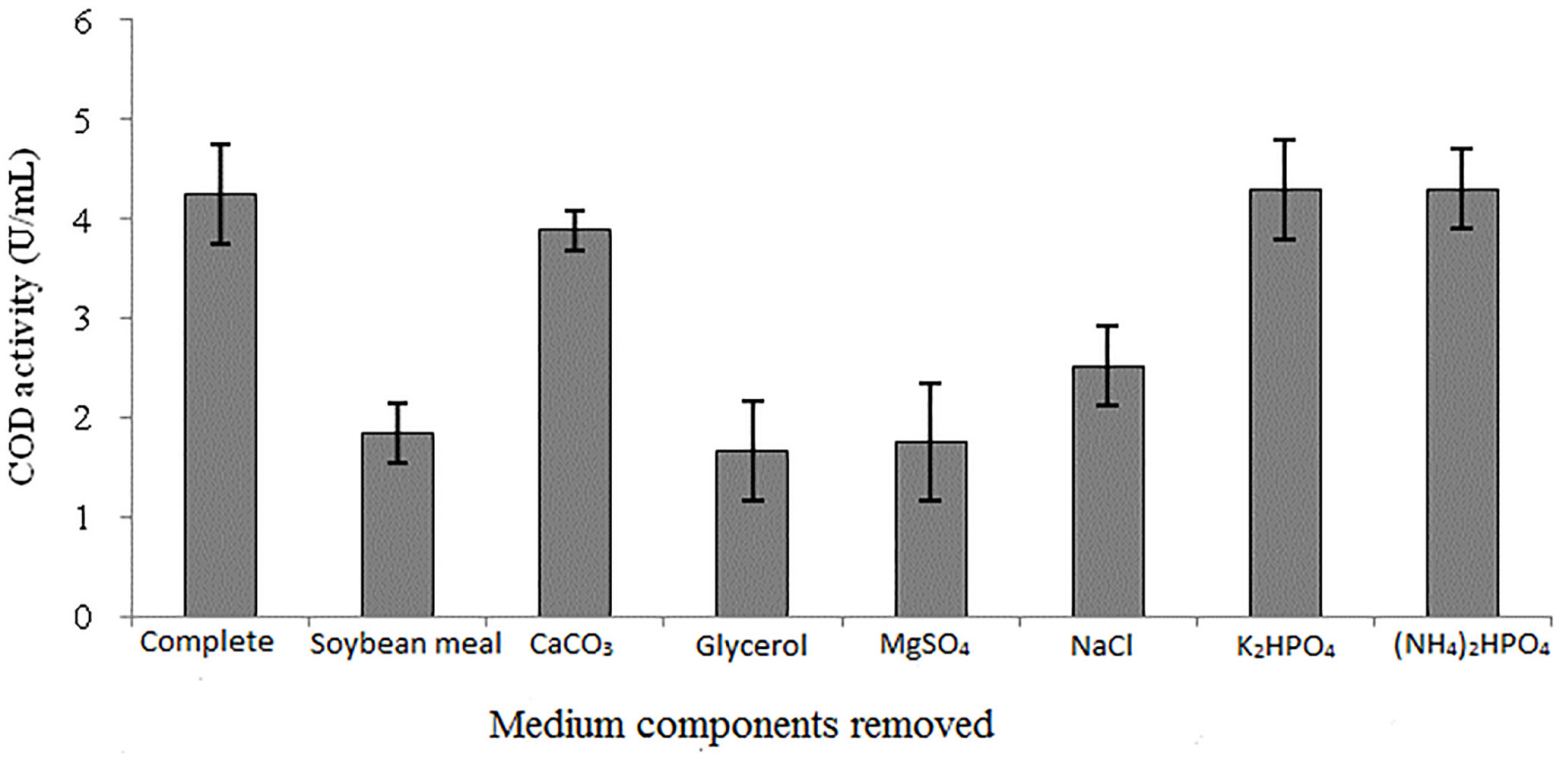
Effect of removal of medium components on COD production.

Soybean meal based X-medium was selected for the production and optimization studies of COD [22]. Under un-optimized production medium conditions, the COD concentration was found to be 4.2 U/mL. In order to enhance the COD production, single-dimension optimization experiments were carried out. The results of removal experiments suggested that removal of soybean meal, glycerol, MgSO_4_, and NaCl shows drastic decrease in COD yield (Fig 1). Further, in carbon and nitrogen supplementation and replacement experiments (Table 2) ammonium ion showed a strong inhibitory action on the COD production, whereas maltose demonstrated positive effect on COD production, hence maltose was included in the statistical medium optimization studies [22].

**Table 2:** CCD (in un-coded) and response values for COD production.

Runs	Soybean meal	Glycerol	Maltose	MgSO_4_	NaCl	Enzyme activity (U/ml)		
						Observed	Predicted RSM	Predicted ANN
1	0.375	0.375	0.375	0.0125	0.225	3.20	2.8171	3.2
2*	0.375	0.375	0.375	0.0375	0.075	2.21	2.02169	2.40
3	0.375	0.375	1.125	0.0125	0.075	1.622	1.76842	0.911
4	0.375	0.375	1.125	0.0375	0.225	3.08	2.71407	3.08
5^*TT*^	0.375	1.125	0.375	0.0125	0.075	2.822	2.93558	2.822
6	0.375	1.125	0.375	0.0375	0.225	3.08	2.08124	3.08
7	0.375	1.125	1.125	0.0125	0.225	2.822	1.6796	2.822
8*	0.375	1.125	1.125	0.0375	0.075	4.752	4.88255	5.80
9	1.125	0.375	0.375	0.0125	0.075	3.08	3.7313	3.522
10	1.125	0.375	0.375	0.0375	0.225	3.78	3.31896	4.77
11^*TT*^	1.125	0.375	1.125	0.0125	0.225	3.08	1.95369	3.08
12	1.125	0.375	1.125	0.0375	0.075	4.708	4.77628	4.708
13	1.125	1.125	0.375	0.0125	0.225	4.80	4.64085	4.8
14	1.125	1.125	0.375	0.0375	0.075	2.048	2.08344	1.94
15	1.125	1.125	1.125	0.0125	0.075	2.268	2.63817	2.1804
16^*TT*^	1.125	1.125	1.125	0.0375	0.225	4.708	4.56582	5.8564
17*	0.0005	0.75	0.75	0.025	0.15	1.222	1.85549	0.8492
18	1.5	0.75	0.75	0.025	0.15	2.926	2.93633	2.9260
19	0.75	0.0005	0.75	0.025	0.15	2.756	3.41425	2.7300
20^*TT*^	0.75	1.5	0.75	0.025	0.15	4.25	4.13571	4.25
21	0.75	0.75	0.0005	0.025	0.15	2.804	2.32791	2.804
22	0.75	0.75	1.5	0.025	0.15	4.365	4.18516	4.365
23	0.75	0.75	0.75	0.0005	0.15	2.282	2.829	2.282
24	0.75	0.75	0.75	0.5	0.15	2.4178	2.92038	2.4178
25*	0.75	0.75	0.75	0.025	0.0005	2.0282	2.74408	3.1949
26	0.75	0.75	0.75	0.025	0.3	2.4178	3.69553	2.4178
27	0.75	0.75	0.75	0.025	0.15	3.08	3.12288	3.181333
28^*TT*^	0.75	0.75	0.75	0.025	0.15	3.282	3.12288	3.181333
29	0.75	0.75	0.75	0.025	0.15	3.182	3.12288	3.181333
30	0.75	0.75	0.75	0.025	0.15	3.282	3.12288	3.181333
31*	0.75	0.75	0.75	0.025	0.15	3.024	3.12288	3.181333
32^*TT*^	0.75	0.75	0.75	0.025	0.15	3.282	3.12288	3.181333
33	0.75	0.75	0.75	0.025	0.15	3.282	3.12288	3.181333
34*	0.75	0.75	0.75	0.025	0.15	3.282	3.12288	3.181333
35	0.75	0.75	0.75	0.025	0.15	2.982	3.12288	2.4178
36	0.75	0.75	0.75	0.025	0.15	3.186	3.12288	3.181333

**Note:** Concentrations of soybean meal, maltose, MgSO_4_ and NaCl are in g/50 ml, whereas glycerol is in ml/50 ml;

* Validation data set

^*TT*^ Testing data set

### Generation of response surface regression model for COD production

After fitting the experimental results in the quadratic (eq 1), the RSM yielded below mentioned response surface model:

$$\begin{array}{l} \text{Y }=\;2.0494\;+\;3.6784{\text{x}}_{1}-0.3495{\text{x}}_{2}-\;3.6367{\text{x}}_{3}-36.0494{\text{x}}_{4}+6.6486{\text{x}}_{5}\;-1.86133{\text{x}}_{1}{\text{x}}_{2}\;+\;\\ 0.040889{\text{x}}_{1}{\text{x}}_{3}-\;8.53333{\text{x}}_{1}{\text{x}}_{4}+\;7.75111{\text{x}}_{1}{\text{x}}_{5}\;+\;0.70222{\text{x}}_{2}{\text{x}}_{3}\;-\;12.26667{\text{x}}_{2}{\text{x}}_{4}\;+\\ \;4.44444{\text{x}}_{2}{\text{x}}_{5}\;+\;136.53333{\text{x}}_{3}{\text{x}}_{4}\;-\;9.68889{\text{x}}_{3}{\text{x}}_{5}\;-\;212.0000{\text{x}}_{4}{\text{x}}_{5}\;-\;1.5605{\text{x}}_{1}^{2}+\;0.9819{\text{x}}_{2}^{2}\;+\\ \;1.1270{\text{x}}_{3}^{2}-\;38.9134{\text{x}}_{4}^{2}\;-\;4.6168{\text{x}}_{5}^{2} \end{array}$$ (2)

Where, Y is the response (i.e., enzyme concentration in U/ml) and X_1_, X_2_, X_3_, X_4_ and X_5_ are the coded values of the test variables, soybean, glycerol, maltose, MgSO_4_ and NaCl, respectively. The goodness of fit of the model is explained by the determination coefficient (R^2^ = 0.920067), which indicates that the second order polynomial model (Eq 2) fits to the experimental data and can explain 92.01% of the variations in the result. The determination coefficient provides the degree of precision of the model in predicting the outcome. Thus, the developed response surface model was capable of predicting the outcomes of the experiment with 92.01% accuracy. The correlation between the independent variables (i.e., medium components) was explained by high value of the correlation coefficient (R = 0.959201). The statistical significance of the second order response surface model was evaluated by ANOVA and F-test. ANOVA validates the fit of the model with the variations observed in the enzyme activity with different variables [25]. The model can be considered significant if the *p*-value ˂0.05 and the F-value should be several times higher than the *p*-value. In this study, high F-value of response surface model with very low *p*-value (F = 71.0678, *p* = 1.251 *10^−7^) shows the statistical significance of the regression model.

The regression coefficient of each variable in terms of linear, quadratic and interaction along with *t*- and *p*-values have been summarized in Table 3. Higher significance of linear, quadratic and their interaction effects of soybean meal, maltose and NaCl (px1 = 0.0123, px3 = 0:0339, px5 = 0.001, px11 = 0.007, px33 = 0.007, px55 = 0.02, px1x2 = 0.0128, px15 = 0.0328, px34 = 0.000005, px45 = 0.0489) than glycerol and MgSO_4_ suggested that they have direct relationship with COD production.

**Table 3:** Regression coefficients for COD concentration.

	SS	Standard Error	MS	F	p
Intercept	0.134323	1.31140	0.134323	0.975958	0.338867
"Var1"	1.112298	1.23221	1.112298	8.081696	0.012344
"Var1"^2	1.294052	0.47078	1.294052	9.402285	0.007838
"Var2"	0.024977	1.23221	0.024977	0.181474	0.676159
"Var2"^2	0.749812	0.47078	0.749812	5.447961	0.03391
"Var3"	1.317302	1.23221	1.317302	9.571211	0.007411
"Var3"^2	0.96088	0.47078	0.96088	6.981531	0.018475
"Var4"	0.105496	30.57876	0.105496	0.766509	0.395103
"Var4"^2	2.201887	14.18936	2.201887	15.9984	0.00116
"Var5"	0.873292	6.16691	0.873292	6.345138	0.023605
"Var5"^2	0.865585	11.79583	0.865585	6.28914	0.024131
"Var1"*"Var2"	1.096209	0.65953	1.096209	7.9648	0.01287
"Var1"*"Var3"	0.000529	0.65953	0.000529	0.003844	0.951384
"Var2"*"Var3"	0.156025	0.65953	0.156025	1.133641	0.30384
"Var1"*"Var4"	0.0256	19.78600	0.0256	0.186004	0.672398
"Var2"*"Var4"	0.0529	19.78600	0.0529	0.384359	0.544583
"Var3"*"Var4"	6.5536	19.78600	6.5536	47.61694	0.000005
"Var1"*"Var5"	0.760384	3.29767	0.760384	5.524774	0.032847
"Var2"*"Var5"	0.25	3.29767	0.25	1.816442	0.197742
"Var3"*"Var5"	1.1881	3.29767	1.1881	8.632459	0.010176
"Var4"*"Var5"	0.632025	98.92999	0.632025	4.592147	0.048926

Var1 = Soybean; Var2 = Glycerol; Var3 = Maltose; Var4 = MgSO_4_; Var 5 = NaCl; SS = Sum of Squares; MS = Mean Square error; F = F-value; p = p-value. Note: The p-values less than 0.05 are significant.

**Fig 2:**
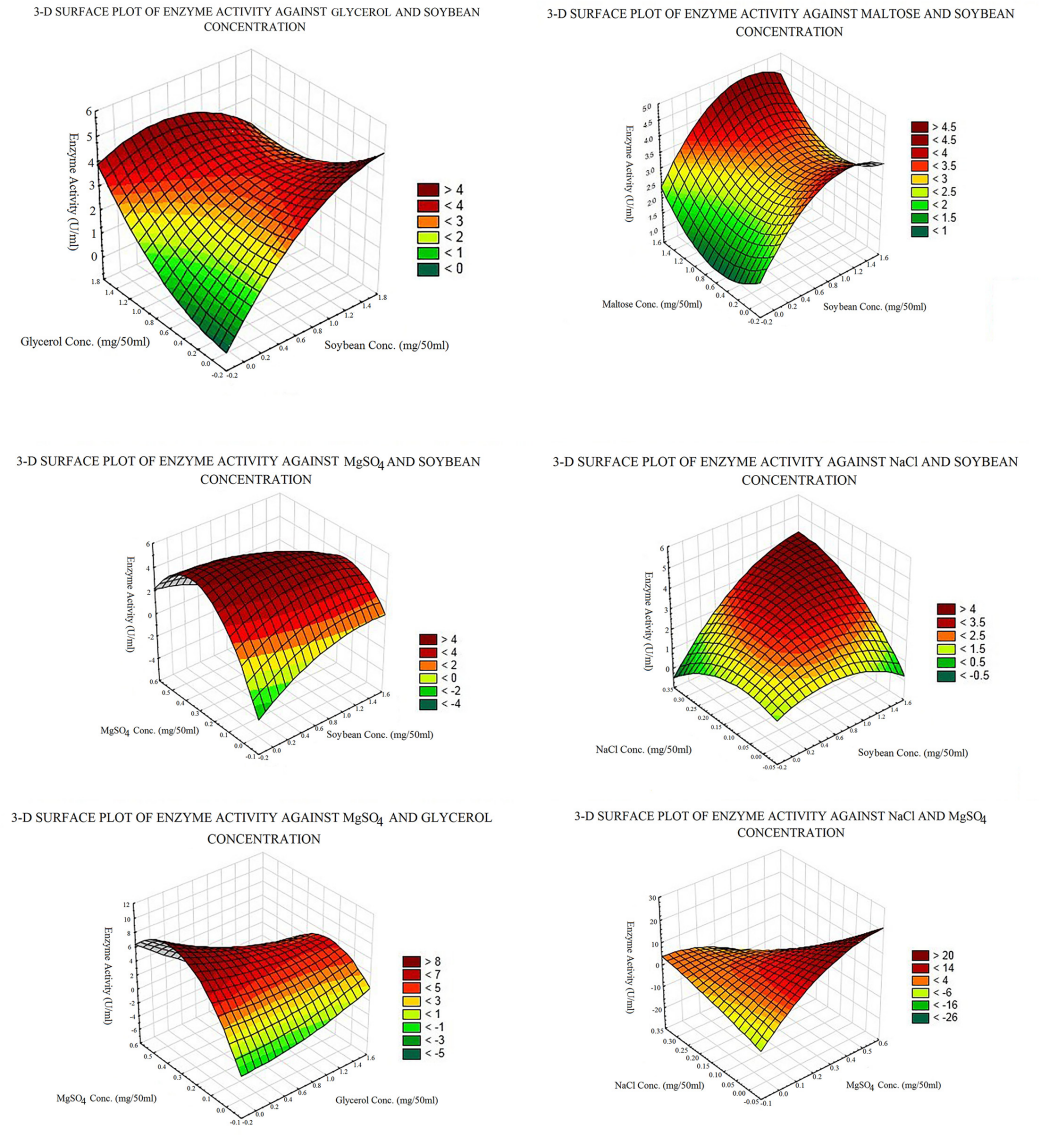
Response surface plots from *Streptomyces* sp. showing effects of medium components on COD production.

Response surface plots (Fig 2) obtained from MATLAB are function of two variables at a time, while maintaining the rest at fixed levels (central values, representing zero level in coded units). Response plots are quite effective in explaining the individual as well as the interaction effects of independent variables (in this case medium components) on dependent variable (Enzyme conc. represented as Enzyme activity) [26]. The dark red regions in each response surface plot represent the regions where maximum enzyme production was observed. It can be observed that soybean and glycerol have an overall weak negative effect on enzyme production. Soybean and maltose appear to have weak positive interaction effect. Soybean and MgSO_4_ show a negative interaction effect increasing both of them together will adversely affect enzyme production. Soybean and NaCl show a strong positive interaction effect. Glycerol and maltose also show a weak positive interaction effect. An interesting observation is a very strong negative interaction effect of Glycerol and MgSO_4_ on the enzyme production. This may be attributed to their specific negative individual effects, which multiplies when these medium components are increased together.

### Generation of ANN regression model for COD production

**Fig 3:**
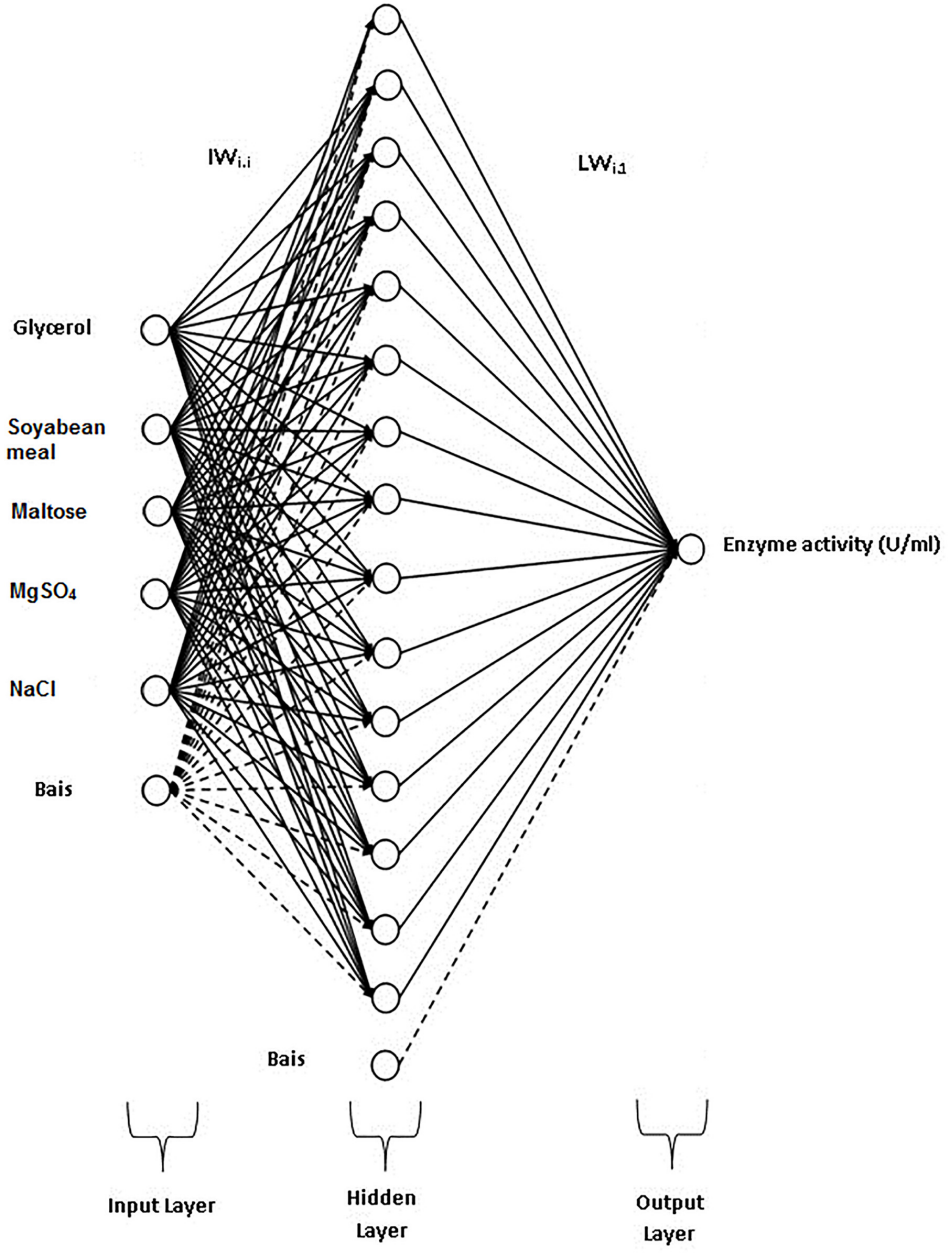
Graphical representation of feed forward neural network containing five components.

A three layered feed forward back propagation neural network having five neurons in input layer and fifteen neurons in hidden layer with hyperbolic tangent sigmoidal transfer function for hidden layer and linear transfer function for both input and output layer was found most efficient and saved (Fig 3). The Levenberg-Marquardt (LM) training algorithm was found to be most accurate and fastest among the three algorithms. The model generated by applying LM algorithm has been given as Eq 3.

$$Enzyme\;activity=\sum_{j=1}^{15}\left\{purelin\left[LW_{J,1}*\left(\sum_{i=1}^{5}\sum_{j=1}^{15}tansig\left(X_{i}*IW_{i,j}+\;b_{j}\right)\right)\right]+a\right\}$$ (3)

**Fig 4:**
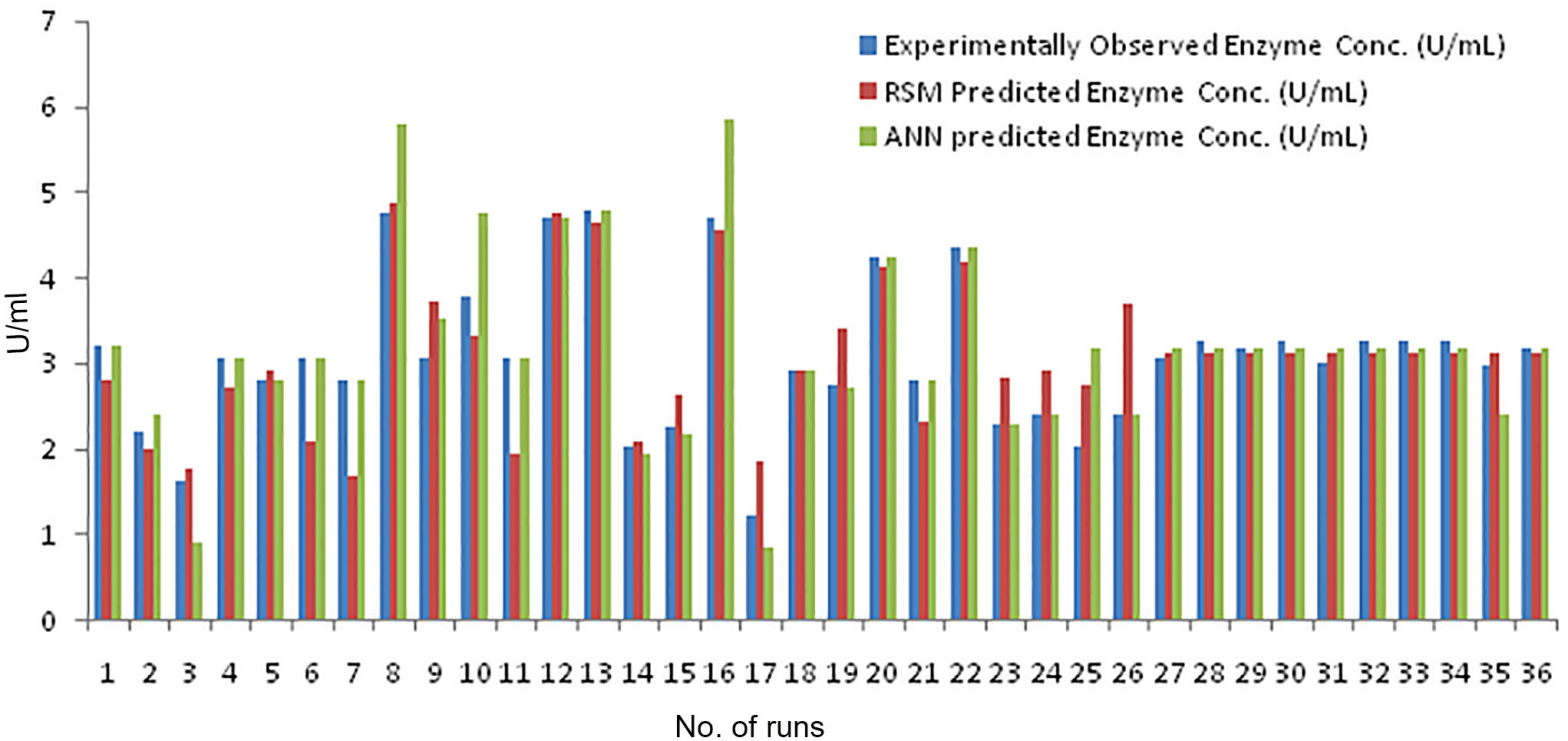
Comparison between observed and predicted enzyme activity from the two models (RSM and ANN).

Eq 3 is the representation of the trained feed-forward ANN model correlating the concentrations of five medium components and the COD concentration in MATLAB. Here, ‘*purelin*’ and ‘*tansig*’ are MATLAB functions which calculate the layer's output from its network input. *purelin* gives linear relationship between the input and the output, whereas *tansig* is a hyperbolic tangent sigmoid transfer function and is mathematically equivalent to ‘*tanh*’. *tansig* is faster than *tanh* in MATLAB simulations, thus it is used in neural networks. LW and IW are weights of connections from the input layer to the hidden layer and from the hidden layer to the input layer, respectively. The weights of *bias* connections of the input and the hidden layers are represented as *b* and *a*, respectively. The input variables have been represented as X. After training the neural networks with LM algorithm, the networks were simulated to predict the enzyme activity for a given media composition. The network learned training data-set with 95.75% efficiency and predicted validation data-set with 93.77% accuracy (Fig 4).

### Optimization of the RSM regression model using GA

**Fig 5:**
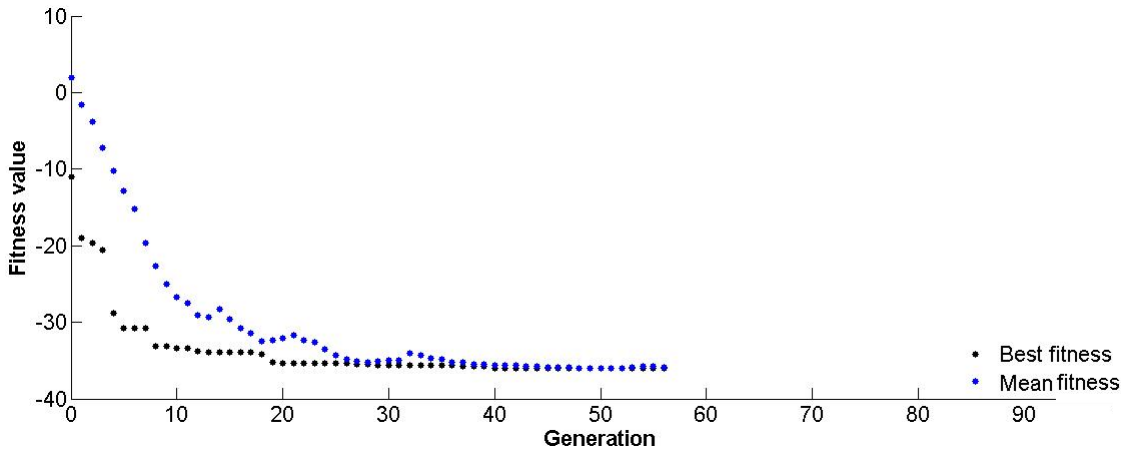
Progressive performance of genetic algorithm generations till optimum solution is achieved.

The final response surface model was optimized using GA. The algebraic form of the model (i.e., Eq 2) was used as a fitness function while performing the optimization by using GA. By employing the defined criteria, the response of the model reaches to its optimum value successfully after eleven generations (Fig 5). The algorithm found maximum output of the enzyme in given experimental bounds at the optimized values of the variables. The maximum enzyme production (6.283 U/mL) was obtained after eleven generations using 1.01 g/50 mL soybean, 1.49 g/50 mL maltose, 0.075 g/50 mL MgSO_4_, 0.45 g/50 mL NaCl and 1.488 ml/50 mL glycerol. However, the GA-optimized (predicted) productivity was verified experimentally and leaded to 6.04 (±0.5) U/mL COD production, which is in close agreement with the GA-predicted COD concentration (6.283 U/mL). Nearly 1.5 folds increase was found in the optimized experimental COD concentration (6.04 U/mL) as compared to the un-optimized medium (4.2 U/mL).

### Optimization of the ANN regression model using GA

The algebraic form of the final trained neural network model (Eq 3) was used as a fitness function of GA to optimize the concentrations of the medium components for maximum COD activity. The model was optimized within the experimental range similar to the optimization of RSM model (Eq 2). Using a population size of 200, the GA reached to the optimum value after 61 generations. Optimization was repeated several times to ensure the global optima. The ANN-GA model predicted a maximum of 9.934 U/mL COD concentration in terms of enzyme activity using 1.431 g/50 mL soybean, 1.389 g/50 mL maltose, 0.029 g/50 mL MgSO_4_, 0.45 g/50 mL NaCl and 2.235 ml/50 mL glycerol. The GA optimized COD concentration was verified experimentally and yielded 9.75 U/mL COD at the optimized concentration. The experimentally verified (media optimized) COD concentration was double (from 4.2 to 9.75 U/mL) than COD concentration obtained with un-optimized medium and nearly 60% higher than the yield predicted by RSM generated model.

## Discussion

Previously it has been reported that COD is the first enzyme involved in the cholesterol degradation and it is produced by various microorganisms. *Arthrobacter*, *Rhodococcusequi*, *Nocardia erythropolis*, *N*. *rhodochrous* and *Mycobacterium* sp. are intracellular/ intrinsic membrane bound COD producers, whereas *Pseudomonas* sp., *Schizopyllum commune*, *Brevibacterium sterolicum*, *Streptoverticillium cholesterolicum*, and some species of *Streptomyces* like *S*. *violascens*, *S*. *parvus*, etc. produces extracellular COD [20, 27–29]. COD produced from *Streptomyces* sp. has been reported to be of higher quality because of lower production cost, stability and longer shelf life [30]. Earlier, we reported extracellular production, purification and characterization of COD by the soil isolate *Streptomyces* sp. NCIM 5500 [22]. We also compared the COD production from free cells to Ca-alginate entrapped cells of *Streptomyces* sp.under batch conditions [31]. However, the production of COD by optimizing the medium components using statistical/ mathematical or artificial intelligence based techniques has not been reported so far from this strain.

Root Mean Square Error (RMSE) and Mean absolute percentage error (MAPE) were determined for the two techniques (RSM and ANN) applied in this study for the prediction of experimentally obtained enzyme concentrations. RMSE and MAPE for RSM are 4.92 and 13.52, respectively, while for ANN they are 4.1 and 7.8, respectively. This qualifies ANN as a better predictor of experimental values as compared to RSM.

The COD production (in terms of enzyme activity) in an un-optimized medium was 4.2 U/ml which was significantly increased to 6.04 U/mL by employing RSM coupled with GA. Whereas ANN coupled with GA resulted in further enhancement in COD concentration (9.75 U/ml,) which was nearly 2.32 folds higher than the yield obtained with un-optimized production medium. A combinatorial method using RSM coupled with GA has been successfully used to solve the problems associated with process optimization [32, 33]. Chauhan et al. (2009) reported 2.48 folds increase in COD productivity from *S*. *lavendulae* by using statistical approaches [34]. Five medium components *viz*. soybean, glycerol, maltose, MgSO_4_ and NaCl found important and were studied for the optimization of COD production. The results of the effect of individual medium component on COD activity correlated to the role of those components for COD production. Glycerol and maltose showed positive effect on COD production. Earlier study also reported that glycerol supports COD production in *S*. *lavendulae* [34]. Soybean meal is a complex nitrogen source and contains amino acids, carbohydrates and also includes fatty acids [4, 34], which enhance the enzyme (COD) production [34]. Here, in this study, during the experiments with *Streptomyces* sp. NCIM 5500, MgSO_4_ was found to be more effective than NaCl for COD activity, which is in contrast to the previous report of Amiri et al. (2008), where they reported NaCl favors COD production than MgSO_4_ [33]. However, other reports support the use of both the salts in the production medium [34]. It was evident from linear and quadratic effect that higher concentration of MgSO_4_ and lower concentration of NaCl is responsible for greater enzyme production. On the contrary to NaCl supplementation in the production medium for COD production, plethora of reports suggests the use of MgSO_4_ for stabilization or even enhancement of COD activity [11, 34, 35]. Also, El- Shoraet al. (2011) reported that COD production activates by Mg^2+^ ions in case of *Staphylococcus epidermidis* [35].

Media optimization using ANN model coupled with GA resulted in higher COD concentration than RSM-GA approach. RSM is a useful technique for understanding the interaction effects of variables but neural network is better in terms of precision, and the same was found in this study. In general, the biological processes are defined by many non-linear complex relationships. ANNs are nonlinear stochastic models that mimic biological neural networks and are efficient in modeling complex biological processes. Desai et al. (2008), compared the efficiency of RSM and ANN in predictive modeling and medium optimization for the production of scleroglucan [36]. They reported that ANN fitted experimental data has greater efficiency than RSM. ANN based model is more generalized as it predicts completely unseen data with greater efficiency (98%) than RSM (89%) [36].

In this study, RSM and ANN were used along with CCD to derive a model for interaction effects of medium components (i.e., soybean meal, glycerol, maltose, NaCl and MgSO_4_) on COD production. Further GA was employed to optimize the RSM/ANN models. The media composition obtained by optimizing both of the models resulted in higher COD concentration than the yield recovered through un-optimized media. This hybrid methodology, i.e., coupling of ANN with GA was found to improve COD production significantly (nearly 2 folds) and proved better than RSM, as the model developed through ANN was found to give nearly 60% higher COD concentration than the yield predicted by RSM generated model. The combinatorial approach (coupling of ANN with GA) presented in this study is sufficiently general and thus can also be successfully employed for the optimization of various parameters used in other bioprocesses. Overall, the higher COD concentration achieved in this study through ANN coupled with GA approach will paves the way for future studies for the production of COD at commercial scale using *Streptomyces sp*. NICM 5500 as well as implication of other/ combination of artificial intelligence techniques for higher and sustainable production of COD.
